# The Anti-Depressant Effects of Statins in Patients With Major Depression Post-Myocardial Infarction: An Updated Review 2022

**DOI:** 10.7759/cureus.32323

**Published:** 2022-12-08

**Authors:** Sai Dheeraj Gutlapalli, Hadi Farhat, Huma Irfan, Kanmani Muthiah, Namratha Pallipamu, Sogand Taheri, Suvedha S Thiagaraj, Twisha S Shukla, Sheiniz Giva, Sai Sri Penumetcha

**Affiliations:** 1 Internal Medicine Clinical Research, California Institute of Behavioral Neurosciences & Psychology, Fairfield, USA; 2 Internal Medicine, Richmond University Medical Center, Staten Island, USA; 3 Cardiology, Rheumatology, California Institute of Behavioral Neurosciences & Psychology, Fairfield, USA; 4 Cardiology, Rheumatology, University of Balamand, Beirut, LBN; 5 Research, California Institute of Behavioral Neurosciences & Psychology, Fairfield, USA; 6 Neurology, California Institute of Behavioral Neurosciences & Psychology, Fairfield, USA; 7 Internal Medicine, California Institute of Behavioral Neurosciences & Psychology, Fairfield, USA; 8 Medical Science, California Institute of Behavioral Neurosciences & Psychology, Fairfield, USA; 9 Pediatric, California Institute of Behavioral Neurosciences & Psychology, Fairfield, USA; 10 Neonatology, California Institute of Behavioral Neurosciences & Psychology, Fairfield, USA; 11 General Medicine, California Institute of Behavioral Neurosciences & Psychology, Fairfield, USA; 12 General Medicine, Chalmeda Anand Rao Institute of Medical Sciences, Karimnagar, IND

**Keywords:** appropriate statin use, statin safety, antidepressant drug, major depression disorder, post-myocardial infarction, hmg- coa reductase inhibitors, statin use

## Abstract

Statins are the most commonly prescribed lipid-lowering agents in patients with cardiovascular disease, and more than half of the patients with cardiovascular disease have associated depressive symptoms, particularly post-myocardial infarction, which is a major trigger for depression. In our research, we tried to understand the anti-depressant effects of statins, the mechanisms, risks and benefits, and potential drug-drug interactions with anti-depressant medications. We reviewed all the relevant information from inception up to September 2022 regarding the anti-depressant effects of statins. The database used was PubMed, and the keywords were statins, major depression, post-myocardial infarction, and hydroxy methylglutaryl-coenzyme A* (*HMG-CoA) reductase inhibitors. We have screened each of the articles carefully, including both human and animal studies, and found a positive correlation between reduction in depressive symptoms with statin therapy as adjunctive treatment with conventional anti-depressants. In conclusion, statins as a monotherapy are not an effective treatment for depression post-myocardial infarction but are good add-on options along with standard therapy such as selective serotonin reuptake inhibitors (SSRIs) and serotonin and norepinephrine reuptake inhibitors (SNRIs). Statins are safe and have no serious drug-drug interactions with anti-depressants. We would like to encourage large-scale observational studies and further post-marketing surveillance to improve our knowledge regarding the effectiveness of statins in the treatment of depression.

## Introduction and background

The prevalence of major depression (MD) in patients with post-myocardial infarction (MI) is a well-known clinical dilemma [1]. Almost 2600 deaths each day are attributed to cardiovascular disease (CVD) in the united states, and more than half of these patients are known to have depression which means almost 1300 deaths occur due to cardiovascular disease in association with major depression [2-7].

Considering the global disease burden due to major depressive disorders (MDD) and non-responders to initial anti-depressant medications, therapies focused on non-monoaminergic pathways for treatment are essential [8]. There is extensive evidence that statins do not lead to depressive symptoms in the general population and may prove beneficial for the treatment of major depression [8].

Our research focuses on the anti-depressant effects of statins in patients with cardiovascular disease and major depression, with a particular focus on post-MI depression. We tried to understand the risks and benefits of statin treatment focusing on its anti-depressant effects and possible drug-drug interactions, and adverse effects on the patient population.

The relevant data for our literature review was gathered from the PubMed database. Four keywords were used "Statins", "Major Depression", "Post-Myocardial Infarction", and "HMG-CoA Reductase Inhibitors", and the search was performed using Medical Subject Heading (MeSH) Strategy. We have carefully screened and included all the relevant articles we could find since inception till September 10^th^, 2022. All data is sourced from PubMed.

## Review

The association between cardiovascular illness and mental health disorders

By the year 2032, heart disease will still be the biggest cause of mortality globally, and depression will become the single largest contributing factor to medical illness leading to long-term disability [5,7,9].

The risk of mental health disorders is significantly higher in patients with pre-existing CVD; MDD is a known trigger of MI, and around two-thirds of the patients with MI have an associated psychiatric illness; furthermore, almost 50% of all CVD patients have depressive symptoms [1,3-7]. There is an obvious bi-directional association between cardiovascular disease and mental health disorders [3,5]. Major depression is reported in 25% of all patients post-coronary artery bypass graft (CABG) surgery [5]. A history of depression increases the risk of acute MI by four times compared to patients without depression. A previous MI is an independent risk factor for new-onset in-hospital depression, which may persist even after discharge [5]. There is around a 20% increased risk of developing heart failure (HF) in patients with depression, and it was also associated with an accelerated progression in patients with pre-existing HF [10].

Diabetes is associated with neurochemical and hormonal changes frequently associated with depression and anxiety [11]. Mental health disorders tripled the risk of medication non-compliance, increased risk of new-onset diabetes, malnutrition, smoking, morbid obesity, alcoholism, sleep disorders, substance abuse disorder, higher frequency of hospitalizations, and increased mortality in patients with cardiovascular disease [5,6,10,12].

Clinical studies evidence that an increased level of pro-inflammatory cytokines is associated with depressive symptoms [13]. Depression is known to promote systemic inflammation, and inflammation promotes fibrosis and adverse ventricular remodeling leading to cardiac dysfunction [10]. Factors like endothelial dysfunction, elevated C-reactive protein (CRP), platelet dysfunction, and reduced flow-mediated vascular dilation, which leads to accelerated atherosclerotic plaque formation in coronary artery disease (CAD) patients, are clearly linked to depression [5,10]. In many cases, a prodrome of minor depression often precedes MI in patients by four to five years [5]. Significantly higher rates of angina, recurrent MI, arrhythmias, and congestive heart failure (CHF) during the initial hospital stay were observed in patients, as well as higher rates of readmissions in patients with depression when compared to non-depressed patients; overall morbidity and mortality in CVD patients were twice as high in depressed vs. non-depressed individuals [5,9]. Studies have shown that patients with distressed type (type-D) personalities are linked to a higher risk of depression and anxiety [6].

**Figure 1:**
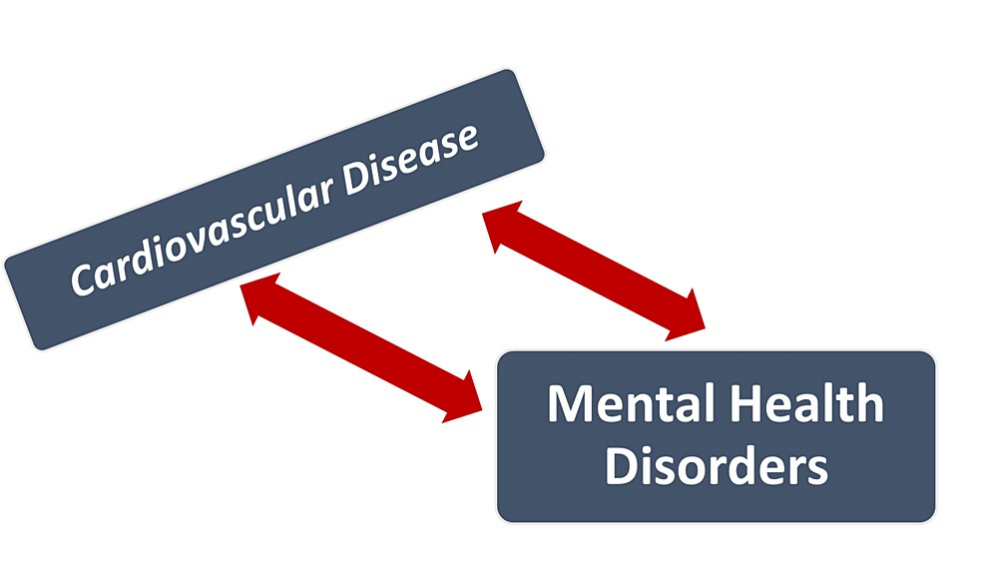
The mutual association between cardiovascular disease and mental health disorders

The relationship between depression and cardiovascular disease is illustrated in Figure 1.

In the United States, almost nine million people have been diagnosed with HF by 2022, with a 50% risk of mortality over the next five years, and more than four million of them have co-morbid depression [6,7,10].

According to the American Heart Association, major depression prevalence is almost three times higher than the general population in patients with acute coronary syndromes (ACS), and the incidence of ACS in patients with depression is thrice that in patients without depression; the prognosis is further worsened by associated serotonin, dopamine, sympathetic and parasympathetic dysfunction in ACS due to co-morbid depression [7,8].

Almost seven million Americans and 16 million people in Europe are currently diagnosed with CHF [12]. Depression triples the risk of development of CHF and doubles cardiovascular, and all-cause mortality compared to the general population, and stifles proper compliance with treatment [12]. The lifetime prevalence of depression in the general population is around 20% across the globe [14].

ACS affects a million Americans every year, with more than half of these patients being previously depressed [15]. Depression, if not treated, has been observed to be stable and unresolving for years post-MI [15]. Patients with major depression who have ACS in-hospital are often reported to be depressed for up to four weeks before the cardiac event in 95% of cases, and 60% of the time patients, were depressed for more than six months before the event [15].

Elevated levels of corticotropin-releasing factor (CRF) in cerebrospinal fluid (CSF) are observed in depressed patients and linked to increased levels of corticosteroids, accelerated atherosclerosis, hypertension, hypertriglyceridemia, and hypercholesterolemia [5]. There is a clear correlation between depression post-MI and level of cardiac dysfunction together with the likelihood of progression to CHF, and major depression is associated with reduced left ventricular ejection fraction (LVEF) 3 to 12 months after MI and LVEF < 30% post-MI has four times higher odds ratio for inciting major depression compared to patients with LVEF > 60% (preserved EF) [12].

In the United States, $50 billion is spent each year on the medical costs related to CHF alone, with a further 30% higher cost of care for patients with associated depression, while in Europe, the cost of care associated with depression is 1% of the overall European Union gross domestic product (GDP) [9,12].

The Johns Hopkins precursor study reported that depression is an independent long-term risk factor for the incidence of CVD and MI. Depression as a single parameter increased the incident risk of CVD by 60% [15]. While looking at myocardial infarction, in particular, depression pre and post-MI significantly worsened medical outcomes compared to non-depressed patients and led to a higher incidence of recurrent adverse events in-hospital [14]. Post-MI depression also increased the risk of mortality by 2.6 times at one-year follow-up and doubled the risk of recurrent MI when compared to non-depressed patients [14]. Of note, the highest risk of adverse outcomes was associated with treatment-resistant depression post-MI [15]. Several reports suggest readmission rates as high as 80% in one-year post-cardiac events in patients with MDD [16]. Patients with post-MI depression and HF have the significantly lower motivation to follow a healthy diet and lifestyle and reduced motivation to complete cardiac rehabilitation [14,17]. The mortality from cardiac causes was two to three times higher than from non-cardiac etiologies in patients with post-MI depression [2].

Statins properties and anti-depressant mechanisms

Statins are 3-hydroxy-3-methyl glutaryl-coenzyme A (HMG-CoA) reductase inhibitors [18]. Statins are cardioprotective due to their potent antioxidant, anti-inflammatory, and lipid-lowering effects [19,20].

Statins are utilized in the prevention of cardiovascular disease primarily due to their effects on cholesterol metabolism; however, statins also have a wider range of effects independent of lipid-lowering mechanisms due to their anti-inflammatory properties [21]. Atorvastatin has neuroprotective and anti-depressant effects [13]. Statins can be divided based on affinity into hydrophilic and lipophilic statins. Lipophilic statins, such as simvastatin, fluvastatin, lovastatin, pitavastatin, and atorvastatin, are more effective at treating depressive symptoms than hydrophilic statins like pravastatin and rosuvastatin [22]. Cholesterol and inflammation are components of neuropsychiatric disorder pathophysiology, and statins, which by inhibition of cholesterol synthesis, are effective in the treatment of dyslipidemia, and CVD and may be effective in curtailing negative neuropsychiatric symptoms in bipolar and other mood disorders [18]. Statins protect against cardiovascular and cerebrovascular disease by effects of cholesterol synthesis and reducing inflammation, thereby, directly and indirectly, affecting the pathophysiology of depression; hence statins are a useful adjuvant therapy to anti-depressants in patients with co-morbid coronary artery disease, diabetes and hypertension [23].

It is clinically proven that the anti-depressant effects of statins are dependent on serotonergic system modulation, which is why they act synergistically with selective serotonin reuptake inhibitors (SSRIs) [24]. Plasma cholesterol levels may be linked to serotonergic neurotransmission and indirectly influence anti-depressant efficacy [25].

In the central nervous system (CNS), proteolytic cleavage of pro-brain-derived neurotrophic factor (pro-BDNF), a precursor of brain-derived neurotrophic factor (BDNF), to BDNF by the tissue-type plasminogen activator (tPA)-plasmin pathway is a major pathway that can control the action of BDNF [20]. Atorvastatin also has anti-depressant effects by regulating neural synaptic plasticity [26]. Atorvastatin also displays anti-depressant and neuroprotective effects against amyloid beta protein 1-40 (Aβ1-40)-induced toxicity, and these effects are linked to tPA- and protein11 (p11)-mediated cleavage of proBDNF to mBDNF [27]. Studies have shown that pro-inflammatory cytokines levels are predictive of the development of depression post-acute coronary syndromes, including myocardial infarction, and statins have significant attenuating effects on inflammatory cytokines, decreasing the incidence of depression [28]. In vitro studies have shown that statins can induce tPA and inhibit plasminogen activator inhibitor-1, which is a major inhibitor of tPA [20]. This mechanism may be a source of the anti-depressant effect of statins [20]. Statins may have potential therapeutic implications in patients with an abnormality in the tPA-plasminogen pathway or other comorbidities relating to cardiovascular disease [20]. Furthermore, Atorvastatin treatment resulted in increased serine/threonine protein kinase (Akt) phosphorylation, synaptic-related protein synapsin, brain-derived neurotrophic factor (BDNF), and spinophilin expression [26]. It was observed that phosphatidylinositol-3 kinase (PI3K) inhibitor LY294002 reversed the Atorvastatin-induced increase of BDNF expression, neurogenesis, and anti-depressant effects [26]. Of note, BDNF dysfunction is also implicated in multiple neuropsychiatric illnesses, such as Alzheimer's disease, Rett syndrome, and attention-deficit hyperactivity disorder [20].

**Figure 2:**
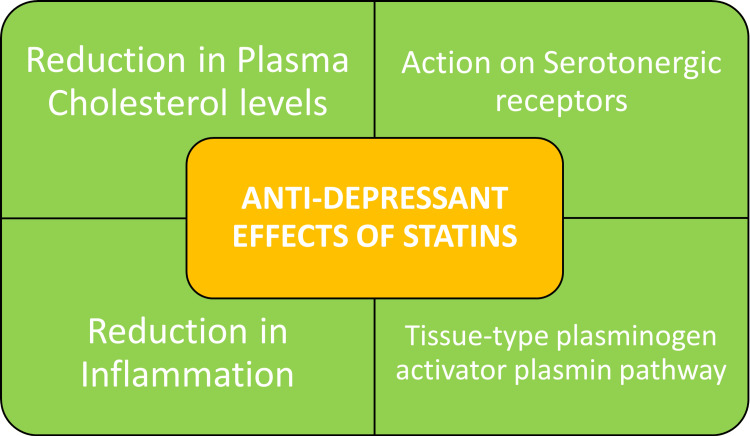
Various mechanisms of the anti-depressant effect of statins

The various mechanisms of statin anti-depressant effects are illustrated in Figure 2.

Statins as add-on therapy for depression

Statins, in combination with SSRIs, have been proven to be generally safe [19]. There is a substantial need for adjuvant treatments to mainstream anti-depressant therapy because the majority of patients have an inadequate response to standard treatments [19].

Statin use in monotherapy does not seem to be effective in the treatment of major depression, but statins may be useful as an add-on therapy along with anti-depressants [29]. Randomized clinical trials have shown that statins have anti-depressant activity when used as add-on therapy, along with SSRIs [21]. Low-grade inflammation is prevalent in depression, hence the utility of statins in its treatment [21]. Increasing central BDNF activity has an important role in the treatment of depression [20]. A wide range of anti-inflammatory drugs have been observed to be useful in patients with MDD and are reasonably safe. Statins are particularly useful given that majority of patients with MDD have co-morbid CVD [30]. Statin therapy is associated with a 32% reduced incidence of depression compared to patients not on statin therapy [31]. A multitude of studies addressing the effects of statins on anxiety, sleep disorders, anhedonia, and psychomotor retardation showed a positive effect with minimal or no negative side effects [8]. Large-scale observational studies have also shown that statins reduce the risk of depression [21]. Statins as add-on therapy to SSRIs are known to have anti-depressant effects in patients with major depression but not in patients without MDD [19].

A Danish nationwide cohort study encompassing 872,216 patients on SSRIs, out of whom 113,108 patients taking statins concomitantly between 1997 and 2012, showed that statin add-on treatment with SSRIs is more effective than treatment with SSRI monotherapy [32]. Double-blinded randomized clinical trials based on patients with moderate to severe depression being treated with citalopram or fluoxetine along with adjuvant statin therapy showed that lovastatin, atorvastatin, and simvastatin improved depressive symptoms [33]. In a one-year follow-up study of a 24-week double-blind, placebo-controlled trial of escitalopram and a prospective observational cohort study, it was indicated that statins, especially lipophilic statins in the treatment of depression post-acute coronary syndromes, are effective add-on treatment options to standard anti-depressant therapy [34]. In a study based on different statins and their effect on depression in post-CABG patients, it was reported that simvastatin had superior anti-depressant effects to atorvastatin [35]. The association between statin therapy and post-stroke depression is still controversial, studies have shown patients receiving pre-stroke statin therapy had a reduced risk of depression, but patients newly started on statin medication post-stroke had a higher incidence of depression [36]. The concomitant use of statins and anti-depressants is seen to reduce the number of major adverse cardiovascular events and depressive symptoms in patients with moderate to high severity of depression but the minimal effect in patients with mild or no depressive symptoms [37].

In a large nationwide cohort study with a 20-year follow-up with a total of around 200,000 statin users and non-users, it was observed that statins treatment was associated with increased anti-depressant use, other prescription drug use, increased risk of depression diagnosis, reduced cardiovascular and all-cause mortality, the higher incidence of depression diagnosis was lost after adjusting for confounding factors [38]. The risk of depression was decreased significantly up to 80% in patients post-myocardial infarction using statins [39]. Of note, Simvastatin is a promising therapeutic option for cognitive disorders with or without focusing on the effects of serum lipids [40]. Prescriptions of anti-depressants and statins should be carefully considered to reduce healthcare costs due to the potentially synergistic nature of the medications in view of depression and cardiovascular morbidity [41]. Particularly, Simvastatin may be useful in patients as an adjuvant medication in treatment-resistant major depression [42]. Clinical trials also suggest that lovastatin as an adjuvant medication is effective for treating patients with MDD [43]. Statins are useful in patients with obesity and co-morbid major depression by the dual effect on depression and cholesterol levels [44]. Statins are also associated with a lower risk of anxiety [45]. Simvastatin may be a good alternative for hormonal replacement therapy in the management of postmenopausal depression without having any deleterious hyperplastic effects on the uterus [46]. In human studies with healthy volunteers, it was observed that atorvastatin had effects on emotional cognitive capacity by increasing the processing of anxiety-associated stimuli like facial recognition, discriminability, and misclassification of fearful expressions [47]. Studies have shown that SSRIs reduce the incidence and progression of atherosclerosis when prescribed along with statins [48].

Many different cardiovascular drugs other than statins have also exhibited anti-depressant effects, including angiotensin-converting enzyme inhibitors, angiotensin II receptor inhibitors, aspirin, and metformin [49].

**Figure 3:**
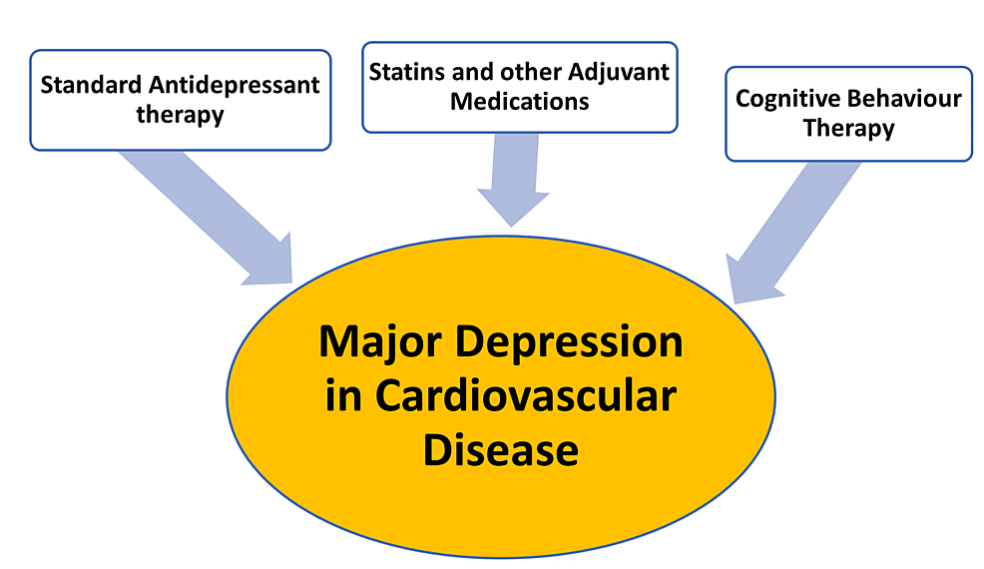
Multi-pronged approach to depression in patients with cardiovascular disease

Figure 3 illustrates the multi-pronged treatment strategy for patients with depression and cardiovascular disease. 

Drug-drug interactions and rare adverse effects of statins

Drug-drug interactions are unlikely to occur as statins are highly selective inhibitors of HMG-CoA reductase with no significant effects on other enzymes or receptor systems [50].

Numerous studies based on anti-depressants like citalopram, escitalopram, mirtazapine, reboxetine, venlafaxine, nefazodone, fluoxetine, paroxetine, and fluvoxamine focused on drug-drug interaction and plasma level of statins due to the effects on the cytochrome enzymes corroborates the general safety profile of statins as adjuvant therapy to anti-depressants [50]. It is common for the elderly to be co-prescribed anti-depressants like SSRIs for psychological conditions and statins for cardiovascular disease prevention. Studies analyzing the risk of drug-drug interactions between SSRIs and statins based on effects on the cytochrome P450 system have shown that citalopram, paroxetine, and escitalopram are safe with all the statins, while rosuvastatin, pravastatin, and pitavastatin are safe with all SSRIs [51].

In a rare case of a patient with idiopathic myelofibrosis and major depression with high cardiovascular risk, cognitive-behavior treatment, and rosuvastatin/ezetimibe were prescribed after discontinuation of atorvastatin and sertraline due to the risk of potential interaction with ruxolitinib, this allowed for continued anti-cancer therapy, and the anti-depressant effect of statin seemed to work in favor of the patient’s psychological improvement along with cognitive behavior therapy (CBT) [52]. Incidents of rhabdomyolysis in association with pravastatin treatment in patients with major depression have been reported [53].

There have been reports of co-prescription of pravastatin and paroxetine leading to an increase in mean serum glucose values and an increase in anti-coagulation parameters like international normalized ratio (INR); these findings may be of concern in certain patients due to increased risk of diabetes, nephropathy, and coagulation disorders [54]. Long-term polypharmacy of statins and anti-depressants may lead to an oxidation-reduction imbalance and increased generation of reactive oxygen species (ROS), inducing cellular dysfunctions, especially in the elderly [55].

Statin use has not been associated with an increased risk of seizures, anxiety disorders, or suicidality [56]. Minimal risk, if at all, for myopathy and rarely rhabdomyolysis is by combining fluvoxamine with simvastatin, lovastatin, or atorvastatin [51]. Rare occurrences of rhabdomyolysis and transaminitis in association with concomitant use of nefazodone and simvastatin have been reported [57].

Relevant information from various animal studies

Animal studies have also shown that statins can potentially reverse the effects of chronic mild stress and associated depressive symptoms [45].

Atorvastatin is shown to have anti-depressant-like effects in animal studies through serotonergic and glutamatergic receptors, the PI3K/Akt/Glycogen synthase kinases-3β (GSK-3β)/mammalian target of rapamycin (mTOR) signaling pathway may also be associated with its anti-depressant effects [8]. Animal studies showed that treatment of atorvastatin (1 to 10mg/kg/day) or fluoxetine prevented lipopolysaccharide (LPS)-induced rise in tumor necrosis factor-α (TNF-α) and lipid peroxidation as well as reduced BDNF levels and glutathione levels in the prefrontal cortex and hippocampus [13]. These effects may be related to effects on TNF-α release, modulation of BDNF expression, and oxidative stress [13]. Animal studies have shown a complete absence of anti-depressant effects in acute atorvastatin treatment with the administration of PI3K inhibitors, rapamycin (mTOR inhibitor), and lithium chloride (GSK-3β inhibitor) [8]. In animal studies, atorvastatin treatment increased the levels of immunocontent phosphorylated isoforms of Akt, GSK-3β, and mTOR in the hippocampus [8]. Animal studies have shown that peroxisome proliferator-activated receptor gamma (PPAR-γ) and nitric oxide systems are important in mediating the anti-depressant effects of atorvastatin [58]. Animal studies show that atorvastatin anti-depressant effects are linked to the inhibition of N-methyl-D-aspartic acid (NMDA) receptors, nitric oxide-cyclic guanosine monophosphate (NO-cGMP) synthesis, and an increase in levels of hippocampal BDNF [59]. Atorvastatin has shown a neuroprotective effect against Aβ1-40 infusion in animal studies [27]. An increase in hippocampal mature brain-derived neurotrophic factor (mBDNF)/precursor BDNF (proBDNF) ratio, which suggests an increase of proBDNF to mBDNF, cleavage is also observed with atorvastatin in animal studies [27]. Increased p11 genic expression and tissue-type plasminogen activator (tPA) have also been observed in the hippocampus of atorvastatin-treated mice [27]. Animal studies have shown that atorvastatin treatment in mice resulted in anti-depressant effects in normal physiological conditions and also in depressive states; these changes depended on α7 nicotinic acetylcholine receptor (α7nAChR) expression in the ventral hippocampus (vHPC) [26].

Simvastatin increased the number of pyramidal neurons in animals on a high-fat diet, showing direct neuroprotective effects [40]. Animal studies have demonstrated that PPARγ receptors and NO-cGMP-ATP sensitive potassium (KATP) channels pathway are involved in the anti-depressant effects of simvastatin [60]. Studies on diabetic rats showed that simvastatin decreased depressive behaviors, increased serotonin concentration in the hippocampus, and decreased corticosterone levels [11]. Simvastatin has anti-anxiolytic effects along with anti-depressant effects, as seen in animal studies [61]. Studies based on a high-fat diet (HFD) and standard diet in animals and the effects of simvastatin showed that simvastatin in controls (standard diet) had anti-depressant, anti-anxiety, and nootropic effects, simvastatin reversed the negative effects of a high-fat diet in test subjects like depressive symptoms, anxiety and reduced cognitive performance [40]. Simvastatin has estrogenic activity and was able to induce the expression of estrogen receptors (ER) and elevation in hippocampal expression of ERα and ERβ along with prohibiting hippocampal microglial activation, abrogated purinoceptor 2X7 (P2X7) receptor, toll-like receptors-2 (TLR2) and toll-like receptors-4 (TLR4) expression, inhibiting activation of nucleotide-binding domain-leucine-rich repeat-pyrin domain containing three (NLRP3) inflammasome, leading to a subsequent reduction in the levels of pro-inflammatory mediators interleukin-1β (IL-1β) and interleukin-18 (IL-18) in animal studies [46].

Low doses of lovastatin have been shown to augment the anti-depressant efficacy of fluoxetine in animal studies [25]. Lovastatin in animal studies has shown significant promise in the treatment of diabetes-associated depression, promoting hippocampal neurogenesis and increased expression of mature brain-derived neurotropic factors [62]. Lovastatin had a significant effect on lipids levels and was compared to a lower effect on blood glucose levels in diabetic animal studies [62].

Animal studies on rosuvastatin showed that two-week simultaneous administration with citalopram resulted in an increase in glutathione peroxidase and glutathione reductase activity but did not change the level of the total antioxidant status [55]. But two-week application of paroxetine significantly decreased glutathione peroxidase activity, with other effects being similar [55].

## Conclusions

Statins have significant anti-depressant effects and are useful as add-on therapy in patients with cardiovascular disease, particularly in post-myocardial infarction patients with major depression. Statins are not useful as monotherapy for depression, the various mechanisms which allow for statins to exert their anti-depressant effects seem to be only clinically effective in the presence of medications such as SSRIs, SNRIs, and other anti-depressants, hence statins would be useful as adjunctive medications and not as monotherapy. The risk of drug-drug interactions and adverse effects with statins and anti-depressant medications is minimal and does not require routine clinical monitoring. Out of all the various statins, simvastatin seems more effective as an anti-depressant add-on, and lipophilic statins have been observed to be superior to hydrophilic statins in their anti-depressant effects. We would also like to encourage large-scale observational studies and further post-marketing surveillance to monitor for any rare adverse events which may occur in individuals with genetic predispositions or acquired conditions.
